# Automated BRDF Measurement for Aerospace Materials and 1D-CNN-Based Estimation of Mixed-Material Composition

**DOI:** 10.3390/s26051560

**Published:** 2026-03-02

**Authors:** Depu Yao, Yulai Sun, Limin He, Heng Wu, Guanyu Lin, Jianing Wang, Zihui Zhang

**Affiliations:** 1 Changchun Institute of Optics, Fine Mechanics and Physics, Chinese Academy of Sciences, Changchun 130033, China; yaodepu21@mails.ucas.ac.cn (D.Y.); sunyulai21@mails.ucas.ac.cn (Y.S.); helimin@ciomp.ac.cn (L.H.); wuheng@ciomp.ac.cn (H.W.); wangjn@ciomp.ac.cn (J.W.); zhangzihui@ciomp.ac.cn (Z.Z.); 2 University of Chinese Academy of Sciences, Beijing 101408, China

**Keywords:** BRDF, aerospace materials, object identification, machine learning, 1D-CNN

## Abstract

With the growing global emphasis on space resources, the significance of space detection and surveillance technologies has escalated. Currently, space-based optical surveillance stands as the primary means for acquiring information on space objects. However, constrained by the diffraction limits of space telescopes, distant space objects are typically imaged as point sources. The resulting lack of sufficient spatial resolution renders traditional image-based recognition algorithms ineffective. In contrast, the Bidirectional Reflectance Distribution Function (BRDF) fully characterizes surface light scattering properties through four-dimensional features, significantly outperforming traditional two-dimensional spectral techniques in material identification. Consequently, leveraging BRDF signatures at varying phase angles has emerged as an effective approach for Space Object Identification. In this study, we developed an automated BRDF measurement system to characterize various typical aerospace materials and investigated the BRDF properties of mixed-material surfaces. A material composition ratio prediction model was constructed based on a One-Dimensional Convolutional Neural Network (1D-CNN). This model effectively extracts key features, including local slope variations and global waveform characteristics, from the BRDF curves. Experimental results demonstrate that the model achieves a maximum relative percentage error of 6.21%, implying a prediction accuracy for mixed-material composition ratios consistently exceeding 93.79%. Compared to image classification methods based on remote sensing imagery, the proposed approach offers higher computational efficiency, significantly reduced model complexity and computational cost, and enhanced robustness. This work provides essential data support for material identification by space-based telescopes and establishes an algorithmic and experimental foundation for intelligent space situational awareness systems.

## 1. Introduction

Since the successful launch of the first artificial satellite, Sputnik 1, by the Soviet Union on 4 October 1957, humanity has successfully sent various spacecraft into Earth’s orbit to explore both the Earth and the space environment [1]. As of 2024, the number of operational spacecraft in orbit globally has surpassed 10,000, reaching a new peak of 11,605, marking a significant milestone in space launch activities [2]. Against the backdrop of an increasingly complex space security environment and deep space exploration missions, high-precision identification of space objects has become one of the core challenges in the development of space technology. From both strategic and commercial perspectives, the high-precision reflective spectral characterization and intelligent identification technology of space objects are regarded as key priorities in the current aerospace field.

Different materials exhibit distinct characteristics across various spectral bands, which are reflected in their unique spectral features. This makes it possible to classify space objects based on their spectral signatures. The precise identification and differentiation of space objects are fundamental to maintaining space security and stability, as well as achieving sustainable space development. Since rocket bodies, debris, and satellites show significant spectral differences, most researchers focus on classifying these three types of space objects [3]. For instance, Song, W. et al. analyzed and processed the scattering spectra of rocket debris and non-functional satellites, finding clear differences in the normalized spectral line shapes between the two categories. They observed that the dispersion difference in normalized spectra for rocket debris was relatively small, while that for non-functional satellites was significantly larger [4]. Bédard et al. conducted continuous observations of the same spacecraft, acquiring multiple sets of measurement data. Based on these measurements, the researchers derived not only scaled relative reflectance spectra but also obtained broadband photometric light curves and color indices [5]. Vananti et al. used a measurement-matching approach to infer the materials of different types of debris. Among them, two types of high area-to-mass ratio debris (gold and silver polyimide) showed a high degree of matching with laboratory-measured spectra [6]. With the rapid advancement of space technology, the increasing complexity of composite materials and hybrid structures used in spacecraft and debris has made material-based identification and differentiation increasingly important. However, due to challenges such as the difficulty in extracting material features, the lack of comprehensive material characteristic databases, and the weak generalization capability of material identification algorithms built on limited prior knowledge, current capabilities in feature extraction and recognition of composite materials remain significantly insufficient. This has led to persistent limitations in recognition accuracy, forming a prominent bottleneck that hinders the improvement of space surveillance effectiveness.

Bidirectional Reflectance Distribution Function (BRDF) measurement, as one of the important means of material characterization, elevates the information dimension from two-dimensional to four-dimensional compared to traditional spectral characterization and identification methods. It can capture the anisotropic reflection characteristics generated by the interaction between light and the microscopic surface structure, thereby addressing the challenge of “different materials exhibiting similar spectral characteristics” that traditional spectral characterization techniques cannot resolve. It also overcomes the difficulty of remotely acquiring information about mixed materials on the surface of space objects using conventional detection methods. A significant advantage of BRDF characterization and identification technology is its ability to identify the material composition of a space object based on its surface BRDF data, even when image recognition technology is not applicable. This validates the feasibility of using space-based telescopes for material characterization and volume estimation of space objects—something that is particularly challenging to achieve with traditional space-based object identification methods. Furthermore, in addition to obtaining material information, in-depth analysis of the BRDF data of space objects can also provide information about their shape, attitude [7], and health status [8].

Due to the difficulty in obtaining actual measurement data of space objects, ground-based measurements of aerospace materials are typically conducted in laboratories to construct spectral databases. These databases are used for comparison and matching with on-orbit data acquired from actual observations, thereby enabling material identification [9]. Sun, C. et al. proposed a five-parameter spectral BRDF statistical model suitable for characterizing surface materials of space objects and performed spectral BRDF measurements on silver foil across the 380–2500 nm wavelength range [10]. Bédard et al. designed and fabricated a fully reflective spectroradiometer to measure the spectral scattering properties of four material samples. Their results demonstrated that different material types exhibit unique spectral bidirectional reflectance distribution functions [11]. Liu, C. et al. introduced an improved BRDF statistical model applicable to modeling multiple types of space object materials. This new model requires only six simple parameters to approximate the surface roughness distribution of materials. Using a genetic algorithm for parameter inversion on 11 different samples of common space object materials, the fitting error for all materials was below 6%, significantly outperforming the five-parameter model [12]. However, studies [10,11,12] only conducted separate measurements and comparisons of individual materials. Research on BRDF measurement, modeling, and inversion under complex mixed-material scenarios remains insufficient, failing to provide the necessary basis for high-precision material identification of space objects.

In summary, this paper designs a measurement system for automatically measuring the BRDF values of material surfaces. The spectral measurement range of this equipment is 400–900 nm. Using this system, systematic absolute measurements were conducted on eight common spacecraft materials: aluminized polyimide films, FEP, double-sided aluminized polyester films, anodized aluminum, black-painted aluminum, white-painted aluminum, solar panels, and circuit boards. This yielded a comprehensive multi-angular and multi-spectral dataset, followed by a comparative analysis of the material characteristics. However, since real-world space targets typically possess multi-material composite surfaces, single-material BRDF data is insufficient for effective identification. Experimental analysis revealed that the BRDF of mixed-material surfaces can be approximated as the linear area-weighted sum of the constituent materials’ BRDFs. Based on this physical assumption, a database comprising 60,000 sets of mixed-material BRDF data was generated by randomly superimposing the values of the eight materials with varying proportions. Subsequently, a machine learning model based on a One-Dimensional Convolutional Neural Network (1D-CNN) was proposed to process this data. The model was trained and validated to predict the fractional composition of constituent materials in new mixed-material samples. Evaluation results indicate that, based on the maximum relative percentage error across the eight materials, the model achieves a prediction accuracy for mixed-material composition ratios consistently exceeding 93.79%. The proposed method, combining BRDF analysis with machine learning, enables the quantitative inversion of surface material composition under unresolved observation conditions, offering a novel pathway for space-based space object identification and characterization.

## 2. Measurement Principles and System Design

### 2.1. BRDF Measurement Principles

**Figure 1:**
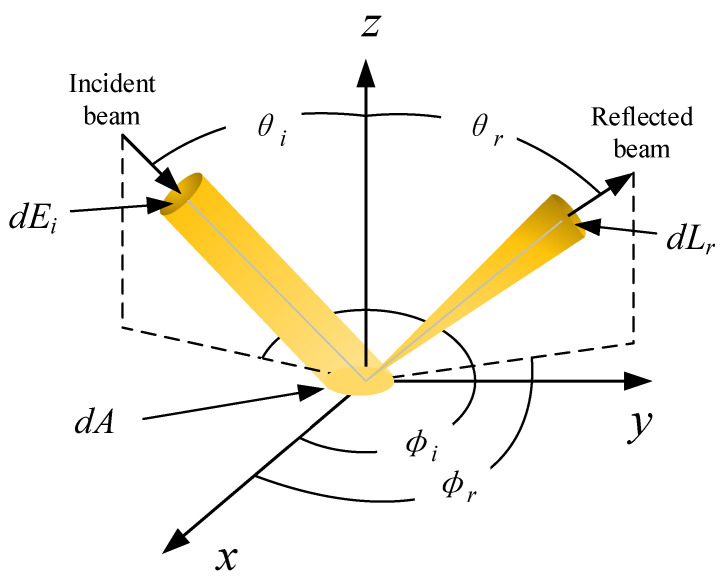
Schematic of the light–surface interaction during reflection.

BRDF is a core theoretical tool for describing the interaction between light and a material’s surface. Since its introduction in the 1970s, BRDF has demonstrated indispensable value in fields such as computer graphics [13], remote sensing science [14], material diagnostics [15], and target extraction and identification [16]. As a common method for characterizing material appearance, BRDF is defined as the ratio of the radiance *dL_r_*, reflected from the surface into a specific direction (*θ_r_*, *φ_r_*), to the irradiance dEi incident from a specific direction (*θ_i_*, *φ_i_*) [17]:

(1) $$f_{r}(\theta_{i},\phi_{i};\theta_{r},\phi_{r})=\frac{dL_{r}(\theta_{i},\phi_{i};\theta_{r},\phi_{r})}{dE_{i}(\theta_{i},\phi_{i})}$$

where *θ_i_* and *φ_i_* represent the incident zenith angle and incident azimuth angle, respectively, while *θ_r_* and *φ_r_* represent the reflected zenith angle and reflected azimuth angle, as shown in Figure 1.

A common simplified form of the BRDF involves assuming the measured object’s surface as an ideal Lambertian reflector. The reflected radiance of an ideal Lambertian reflector depends solely on material properties and incident irradiance, independent of the angles of incidence and reflection. Although this assumption is frequently adopted in many scenarios, only a very limited number of material surfaces in practice meet this criterion. For relatively smooth surfaces, a significantly enhanced specular reflection component is observed when the incident zenith angle *θ_i_* equals the reflection zenith angle *θ_r_* and the azimuth angles differ by 180°. Conversely, for relatively rough surfaces, reflection enhancement also occurs in the incident direction (where *θ_i_* = *θ_r_* and *φ_i_* = *φ_r_*). In the field of space situational awareness, accurate optical identification of spacecraft, space debris, and other targets relies heavily on an in-depth understanding of their visible/infrared reflection characteristics. Traditional simplified reflection models, such as the assumption of Lambertian surfaces, fall short of meeting the demands for high-precision identification. Therefore, constructing and updating a BRDF database for aerospace materials is a fundamental and indispensable step toward overcoming the bottlenecks in optical identification of space targets, playing a critical role in space object recognition.

### 2.2. BRDF Measurement System

BRDF is a crucial factor describing the optical properties of a material surface. However, even for simple surfaces, measuring the BRDF is highly complex. As the function’s value varies with the zenith and azimuth angles of both incidence and reflection, characterizing the BRDF requires a large number of measurements at different angles and positions on the target material surface. Hence, this paper presents the design of an automated BRDF measurement system developed to measure the BRDF values of various aerospace material surfaces.

**Figure 2:**
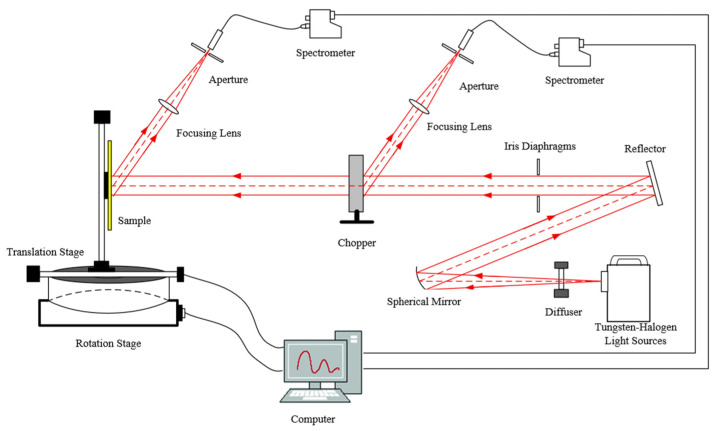
Diagram of the BRDF measurement system.

The apparatus consists of a collimated broadband light source system, a spectroscopic monitoring system, a motion control system, and a main optical path measurement system. Since reddening is a prevalent phenomenon at wavelengths greater than 600 nm—meaning that for the same surface, the reflectance measured in actual observations is generally higher in the longer wavelength range compared to ground-based experimental results, with the deviation increasing as the wavelength increases—the spectral measurement range of this device is selected as 400–900 nm, with a spectral resolution better than 5 nm. The incident zenith angle *θ_i_* can be varied from 0° to 85°, and the reflected zenith angle *θ_r_* from −85° to 85°. The incident azimuth angle *φ_i_* is adjustable within 0–45° (specifically at 0°, 15°, 30°, and 45°), while the reflected azimuth angle *φ_r_* covers 95–265°. The angular resolution is less than 0.05° with good repeatability. The measurement principle is illustrated in Figure 2.

**Figure 3:**
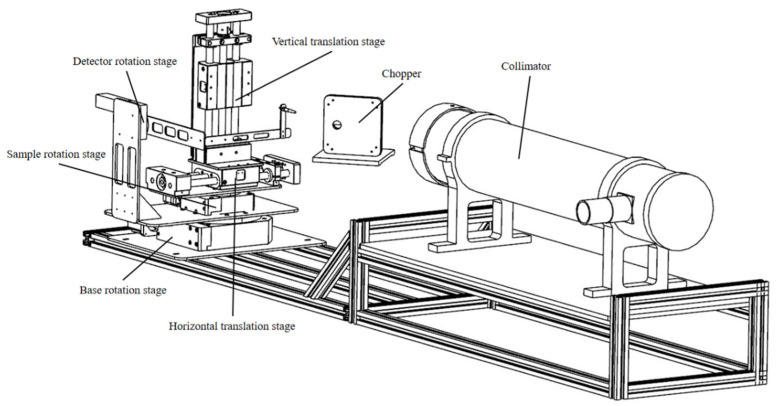
Diagram of the measurement system structure.

The design scheme of this measurement system is based on the theoretical foundation of the light source monitoring ratio compensation method and the structural framework of the optical path, with its schematic diagram shown in Figure 3. A halogen tungsten lamp serves as the light source, which is collimated through a parallel optical tube system. The collimated light is then split by a chopper modulation wheel, resulting in two mutually perpendicular beams of parallel light: one beam continues as the main optical path and irradiates the surface of the sample under test, with the reflected spectral information from the sample surface collected by the main optical path spectrometer; the other beam serves as the monitoring optical path and is directly received by the monitoring optical path spectrometer.

#### 2.2.1. Light Source

To obtain accurate BRDF values of the measured object, it is essential to ensure stable and uniform illumination on the object’s surface. There are two basic optical designs for surface measurement. The first involves irradiating a specific small area on the surface using a light source, while an observation instrument monitors the entire measured surface. The second approach entails illuminating a large area of the surface and using the instrument to observe only a portion of the irradiated region. The first method imposes strict requirements on the distance between the optical fiber interface and the measured surface, as an increase in distance leads to a larger observed area, thereby reducing the proportion of the irradiated region within the field of view and affecting measurement accuracy. In contrast, the second method is less sensitive to changes in the distance between the optical fiber interface and the measured surface, albeit with some loss of incident light efficiency. Therefore, the second illumination scheme was selected.

**Figure 4:**
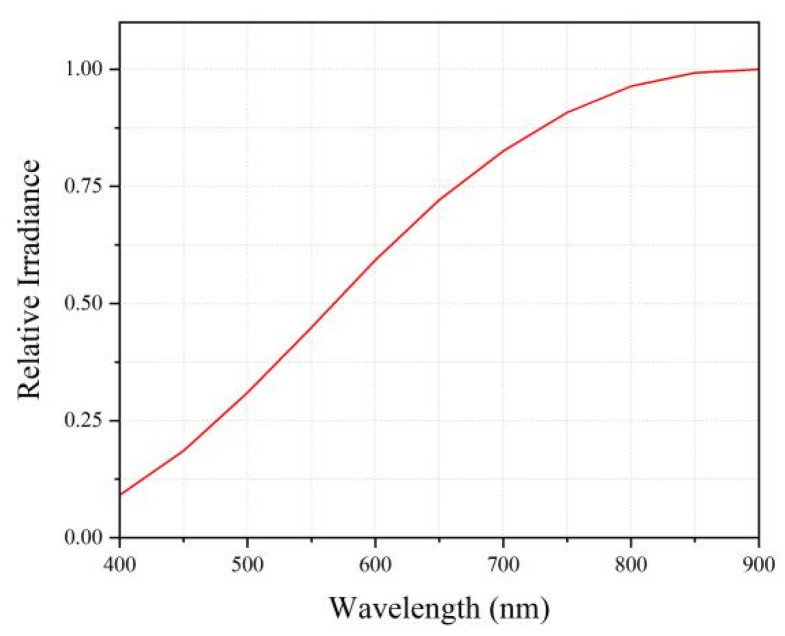
Relative Irradiance of the Light Source.

A halogen tungsten lamp was selected as the light source, offering spectral coverage from visible to near-infrared light, thereby fully ensuring a broad spectral range. Additionally, its advantages of high intensity and high luminous flux reduce the performance requirements for the detector and facilitate the acquisition of experimental data. Other benefits include low cost, ease of operation, no need for complex drive circuits, and no requirement for preheating. Although halogen tungsten lamps have drawbacks such as relatively low efficiency and significant heat generation, their exceptional broadband continuous output, high brightness, and strong near-infrared radiation capacity make them indispensable for simulating sunlight or conducting broad-spectrum analysis. To obtain the high-quality collimated light required for the experiment, the light beam is first homogenized and then collimated through a collimating tube. An adjustable aperture with a diameter range of 2.5–42 mm is installed at the output end of the collimating tube to ensure that the emitted collimated light passes smoothly through the detection optical path. The relative irradiance of the light source used is shown in Figure 4. Long-term stability tests of this light source system confirmed that the output intensity fluctuation remains within ±2%.

#### 2.2.2. Beam-Splitting Monitoring System

The spectroscopic monitoring system employs a modulation wheel functioning in a semi-transparent and semi-reflective manner as the beam-splitting element. The modulation wheel features equal areas of transparent and reflective regions. Based on the output beam aperture of the light source system, the modulation wheel is designed with a diameter of 100 mm, and its effective modulation area diameter exceeds 40 mm. The modulation wheel utilizes an aluminum substrate, which undergoes optical polishing followed by nickel plating to enhance surface flatness and reflectivity stability, thereby ensuring measurement consistency in the monitoring optical path. Driven by a DC brushless motor, the modulation wheel achieves beam splitting through periodic switching between its reflective and transparent regions. Experimental measurements indicate that the reflectivity of the modulation wheel surface is 79%, and the incident energy levels for both the monitoring system and sample measurements are comparable, meeting the system’s monitoring requirements.

#### 2.2.3. Mechanical Motion System

The mechanical motion system comprises three motorized rotation stages and two motorized translation stages. It enables the measurement of the BRDF values for different areas of the material under test, as well as for various incidence and reflection directions, through coordinated multi-degree-of-freedom movement. The measurement system possesses a total of five degrees of freedom: the rotation angles of the base rotation stage, sample rotation stage, and detector rotation stage are denoted as α, β, and γ, respectively, with α ∈ [0°, 180°], β ∈ [−90°, 90°], and γ ∈ [−90°, 90°]. The clockwise direction is defined as positive for rotation. The displacements of the horizontal translation stage and vertical translation stage are denoted as x and y, respectively, with x ∈ [−50 mm, 50 mm] and y ∈ [−50 mm, 50 mm]. The rightward horizontal direction and upward vertical direction are defined as positive for translation. Repeatability tests have verified that the positioning accuracy of the motorized rotation stages is better than 0.05°, and the positioning accuracy of the motorized translation stages is better than 0.1 mm.

The displacements x and *y* determine the two-dimensional coordinates of the measurement point on the surface of the test sample. By selecting multiple different target points for measurement, the random errors caused by local inhomogeneities can be effectively mitigated. The rotation angles α, β, and γ are used to control the zenith and azimuth angles of the incident and reflected light. This enables the measurement of the sample’s BRDF values under various geometric conditions, thereby allowing for a comprehensive and accurate characterization of the spatial reflection properties of the sample surface.

#### 2.2.4. Detector

This experiment utilizes a dual-spectrometer configuration for spectral BRDF measurements. The selected spectrometers are equipped with silicon-based linear array CCD detectors, which offer a signal-to-noise ratio of up to 250:1 and allow the integration time to be adjusted within a range of 3.8 ms to 10 min. The instruments cover a spectral range of 350–1100 nm with a spectral resolution better than 5 nm. Reflected signals from the sample surface are collected by a fiber optic probe equipped with an aperture. This layout effectively avoids interference with the movement of the motorized rotation stages, significantly enhancing the flexibility and adaptability of the measurement system. During the alignment process of the fiber optic probe, it is replaced by a visible laser source. By adjusting the position and orientation of the laser, the laser spot is ensured to accurately cover the target area on the sample surface.

#### 2.2.5. Design Error Analysis

The errors in BRDF measurement results arise from multiple factors, primarily attributed to mechanical motion inaccuracies, light source system errors, detector errors, and human factors [18]. This error *ε_BRDF_* can be expressed by the following formula:

(2) $$\varepsilon_{BRDF}^{2}=\varepsilon_{M}^{2}+\varepsilon_{I}^{2}+\varepsilon_{R}^{2}+\varepsilon_{P}^{2}$$

where *ε_M_* denotes the mechanical motion error, *ε_I_* represents the light source system error, *ε_R_* signifies the detector error, and *ε_P_* corresponds to the human-induced error.

The mechanical motion error primarily comprises the positioning errors of the rotation and translation stages. The three rotation stages used in this experiment have a motion error of less than 0.005°, while the two translation stages have a motion error of less than 0.05 mm. Since the translation stages remain stationary during the measurement of BRDF values at fixed incidence angles, their associated errors are not included in the calculation. The overall mechanical motion error is determined to be less than 0.87%.

The illumination system error primarily arises from source intensity fluctuations and stray light. Experimental results indicate that the intensity fluctuation is less than 2%, and stray light was controlled within 1% during the measurements. Consequently, the total illumination system error is calculated to be less than 2.23%.

The detector error primarily includes contributions from the signal-to-noise ratio, detector nonlinearity, and the fiber optic’s solid angle error. The error induced by the SNR is 0.4%, the nonlinearity error is approximately 0.6%, and a *Z*-axis alignment error of the optical fiber of 0.2 mm results in a solid angle error of 0.4%. The total detector error is calculated to be less than 0.83%.

The human-induced error, attributed to uncertain factors, was estimated to be less than 4.5% based on the comparison of repeated measurement results.

Substituting the aforementioned data into Equation (2) yields a total system error of less than 5.16%.

To validate the practical measurement error and repeatability precision of the system, five repeated experiments were conducted on the same material under identical conditions, with 20 sets of spectral data acquired in each experiment. Statistical analysis was performed on the 100 collected samples, and the variation in the quantitative results was confined within 4.6%, indicating that the proposed experimental system exhibits good repeatability and stability. This level of variation demonstrates that the effects of random errors and measurement uncertainties are effectively controlled, thereby confirming the reliability of the experimental results.

### 2.3. A 1D CNN-Based Method for Predicting the Composition Ratio of Mixed Materials

A Convolutional Neural Network (CNN) is a multi-layer supervised learning neural network, typically comprising convolutional layers, pooling layers, activation layers, and fully connected layers. Its training method involves minimizing the network’s loss function through gradient descent to obtain the weight parameters in the convolutional layers [19]. The convolutional layer serves as the core component, performing convolution operations on the input data via a series of filters (kernels) to extract specific local features from the data. Unlike fully connected layers, each neuron in a convolutional layer is only connected to a local region of the previous layer, significantly improving computational efficiency. The pooling layer, usually placed after the convolutional layer, is employed to downsample the feature maps. This serves to reduce computational load and the number of parameters, lower data dimensionality, control overfitting, and enhance the model’s robustness to minor variations. The activation layer introduces non-linearity into the network, enabling the neural network to approximate any complex function. The input to the fully connected layer consists of feature maps obtained after feature extraction by the convolutional and pooling layers, and it is used for the final classification or regression output. The last layer of the network is the output layer [20].

Researchers have proposed various variants based on two-dimensional convolutional neural networks (2D-CNNs) to handle one-dimensional data for the following reasons:

1. Forward Propagation (FP) and Backpropagation (BP) are the two core processes in neural network training, working together to enable the learning of mappings from input to output. Owing to the involvement of matrix operations, the computational complexity of a one-dimensional convolutional neural network (1D-CNN) is significantly lower than that of a 2D-CNN;
2. Compared to the deep architectures typically employed in 2D-CNNs, the shallower architecture of 1D-CNNs is easier to comprehend, train, and implement, making it more suitable for learning from one-dimensional data;
3. 1D-CNNs are capable of processing data utilizing substantially fewer computational resources, whereas 2D-CNNs impose higher demands on hardware [21].

The 1D-CNN is a natural extension of the CNN, adapting the convolution operation from the two-dimensional image space to the one-dimensional sequence space, where its filters perform convolutions along a single dimension. It can efficiently capture local features within spectral signals and progressively integrate these local features into more global patterns across layers. With faster training speed and a lower risk of overfitting, the 1D-CNN is particularly suitable for scenarios with limited data availability and offers unique advantages for the extraction and identification of mixed-material BRDF values. Therefore, this paper adopts the 1D-CNN model for the identification of mixed-material BRDF values.

**Figure 5:**
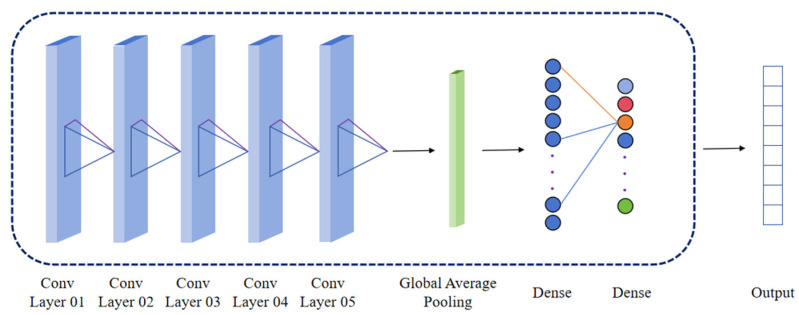
Proposed 1D-CNN Architecture.

Based on a 1D-CNN, this study proposes a machine learning model for processing mixed-material BRDF data. As illustrated in Figure 5, the model comprises an input layer, five convolutional layers, a global average pooling layer, two fully connected layers, and an output layer. The five convolutional layers are designed to extract complex features ranging from low-level to high-level representations. They employ 64, 128, 256, 512, and 512 filters, respectively, progressively enhancing the model’s representational capacity. The kernel size is 5 for the first layer and 3 for the subsequent layers. The Rectified Linear Unit (ReLU) is used as the activation function. Each convolutional layer is followed by a dropout layer with a rate of 30% to prevent overfitting. A global average pooling layer is incorporated after the convolutional blocks. This layer replaces the traditional flattening operation by computing the average value for each feature map, thereby reducing the number of parameters and improving the model’s generalization capability. The two fully connected layers contain 256 and 128 neurons, respectively. They also utilize the ReLU activation function. The dropout rate is increased to 50% for these layers due to their higher susceptibility to overfitting. The output layer uses the Softmax activation function to ensure the outputs sum to 1, making it suitable for proportional prediction tasks. A summary of the model’s architecture is provided in Table 1.

This model treats BRDF data as a signal sequence possessing local correlations. Through its deep architecture with five convolutional layers, it is capable of automatically learning hierarchical features, progressing from simple reflectance variations to complex mixtures of material appearance. The model is optimized in terms of both parameter count and structure to effectively prevent overfitting. By modifying the final fully connected and output layers, it can be adapted to different tasks. This model represents a highly rational and superior choice for high-dimensional, structured, sequential data with local correlations, such as BRDF data. It significantly outperforms traditional fully connected neural networks in retaining critical information, mitigating overfitting risks, and enabling automated feature learning.

## 3. Experimental Results

### 3.1. BRDF Measurement Results

The measurement experiment selected eight materials commonly used in spacecraft: aluminized polyimide film, FEP, double-sided aluminized PET film, anodized aluminum, black painted aluminum, white painted aluminum, solar panel, and circuit board. With the exception of the solar panels and circuit boards, each sample measured 150 mm × 150 mm and could be mounted on the surface of a vertical translation stage using an adapter plate.

The measurement system designed in this study supports both absolute method-based measurements and relative measurements using a standard diffuse reflector with known BRDF values. For this particular experiment, the absolute measurement method was selected. Based on the BRDF measurement principle described previously, the incident spectral irradiance and reflected spectral radiance were measured separately. The BRDF value of the sample under test can then be calculated using Equation (1). Herein, the incident spectral irradiance can be derived from the incident power of the light source and the illuminated area on the sample surface, while the reflected spectral radiance can be calculated from the reflected spectral power, the detector’s solid angle, and the acceptance area.

**Figure 6:**
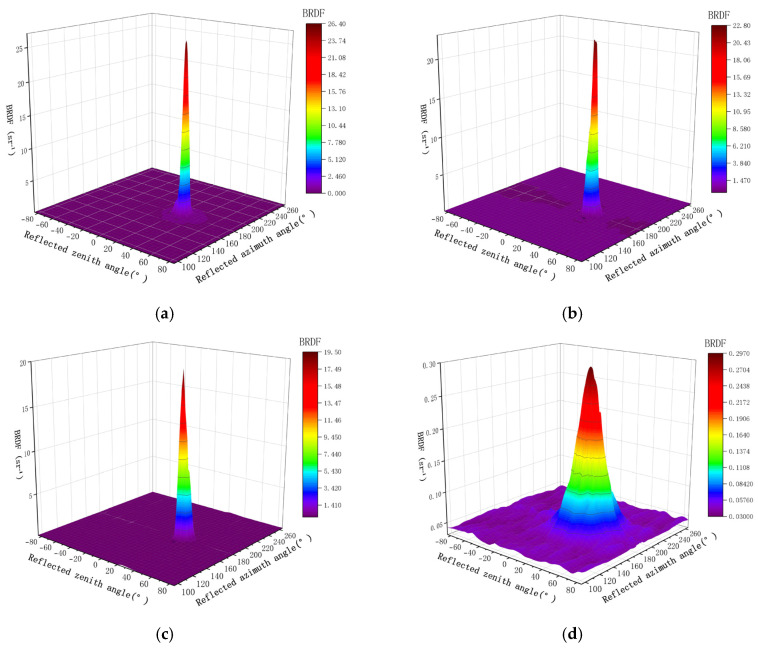

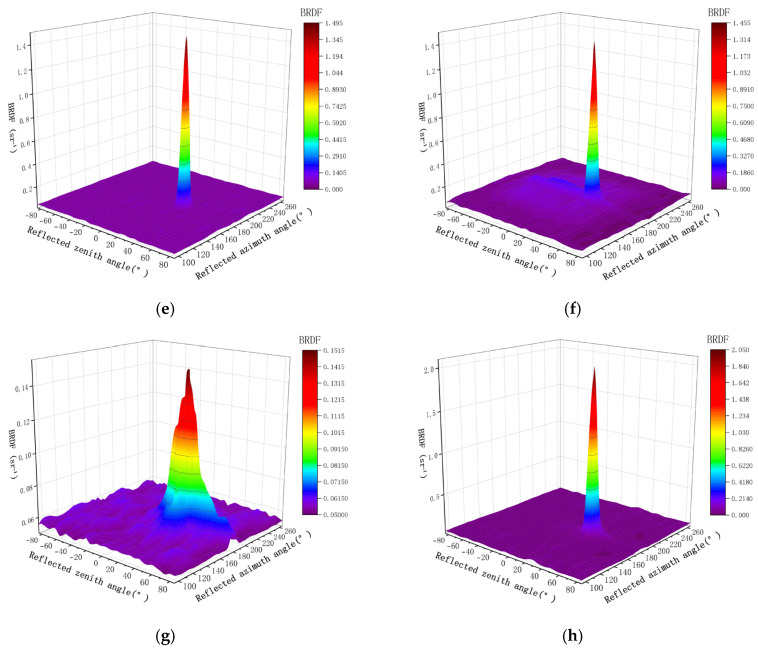
BRDF Values of the Eight Materials at 700 nm (*θ_i_* = 30°, *φ_i_* = 0°), (**a**) aluminized polyimide film, (**b**) FEP, (**c**) double-sided aluminized PET film, (**d**) anodized aluminum, (**e**) black painted aluminum, (**f**) white painted aluminum, (**g**) solar panel, (**h**) circuit board.

For any given incident zenith angle *θ_i_* and incident azimuth angle *φ_i_*, the measurement interval was set to 2° when the reflection zenith angle fell within the range of *θ_i_* − 10° ≤ *θ_r_* ≤ *θ_i_* + 10°, and to 5° when *θ_r_* < *θ_i_* − 10° or *θ_r_* > *θ_i_* + 10°. Similarly, for the reflection azimuth angle, a 2° interval was used within *φ_i_* + 170° ≤ *φ_r_* ≤ *φ_i_* + 190°, and a 5° interval was applied when *φ_r_* < *φ_i_* + 170° or *φ_r_* > *φ_i_* + 190°. The BRDF values of the eight materials, measured at an incident zenith angle *θ_i_* = 30°, an incident azimuth angle *φ_i_* = 0°, and a wavelength of 700 nm, are presented in Figure 6.

**Figure 7:**
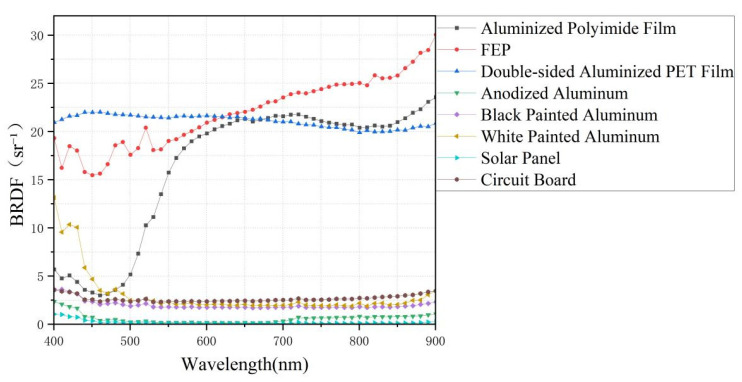
BRDF Values of the Eight Materials over the 400–900 nm Wavelength Band.

As observed in the figure, the maximum BRDF values occur near the specular reflection direction, irrespective of the material surface roughness. Moreover, materials with stronger specular reflection exhibit sharper peaks in their BRDF profiles. aluminized polyimide film, FEP, and double-sided aluminized PET film show significant BRDF peaks, which can be attributed to their low surface roughness and consequently weaker scattering effects. However, due to the susceptibility of these three materials to surface wrinkles, variations in BRDF values may occur if the detector’s measurement area is small. This necessitates measurements at multiple positions during experiments to exclude data outliers. In contrast, the anodized aluminum sample, having undergone sandblasting, exhibits pronounced scattering, resulting in a lower BRDF peak and a slower angular decay rate compared to the other materials. The BRDF characteristics of black painted aluminum and white painted aluminum at 700 nm are generally similar, with only minor differences observed near *φ_r_* = 180°. The solar panel exhibits a relatively low BRDF peak of only 0.15 at 700 nm due to its inherent spectral absorption properties. Additionally, experimental results indicate that the BRDF value of a circuit board is influenced by the number of surface pads and electronic components; thus, variations may exist between different circuit boards (the circuit board shown in Figure 6 represents one of the test samples). The data in Figure 6 can only reflect the characteristics of one material at a fixed wavelength. To identify mixed materials, BRDF values of the materials at multiple bands are required. Figure 7 shows the BRDF values of eight materials in the 400–900 nm band (*θ_i_* = *θ_r_* = 20°, *φ_i_* = 0°, *φ_r_* = 180°), which can more intuitively reflect the changing trend of BRDF values of different materials within a relatively wide band, facilitating the extraction and identification of BRDF values of different components in mixed materials. Table 2 presents the key statistics of the eight materials across the entire wavelength range. Although some materials show similar trends in certain wavelength bands, the overall characteristics of these eight materials are quite distinct, which facilitates the subsequent extraction and identification of mixed BRDF values.

### 3.2. Prediction Results of Mixed-Material Component Proportions

Measurements from multiple mixed-material samples indicate that the BRDF value of a composite material can be approximated as a linear area-weighted sum of the constituent materials’ BRDFs. While mutual shadowing and multiple scattering occur at material boundaries, the surface area within each material patch significantly exceeds the boundary area. Consequently, these non-linear edge effects contribute negligibly to the total radiant flux and can be disregarded as second-order terms. To validate the efficacy of the proposed machine learning method, we utilized BRDF data acquired at fixed incident and viewing angles. The measurement geometry was set to *θ_i_* = *θ_r_* = 20° and *φ_i_* = 0°, *φ_r_* = 180°. After securing each material sample, 10 measurement points were randomly selected via horizontal and vertical translation stages. Each point was measured 100 times, yielding 8000 sets of raw BRDF data across the eight material types. The data covers a spectral range of 400–900 nm with a resolution of 10 nm, comprising 51 spectral points per dataset. To construct the dataset, 1 to 8 material types were randomly selected. For each type, one spectral curve was drawn from its 1000 raw measurements. These were combined using mixing proportions generated via a Dirichlet distribution to ensure the coefficients sum to unity. To simulate realistic observation conditions, 3% Gaussian noise was injected into the synthetic data. In total, 60,000 mixed-material samples were generated. The dataset was partitioned into a training set (70%, 42,000 samples), a validation set (15%, 9000 samples), and a test set (15%, 9000 samples). A fixed random seed was employed to ensure deterministic dataset generation and experimental reproducibility; altering the seed allows for the generation of varied datasets. This partitioning strategy adheres to standard machine learning practices, ensuring that the model is trained, validated, and tested on independent subsets to yield a reliable performance evaluation.

**Figure 8:**
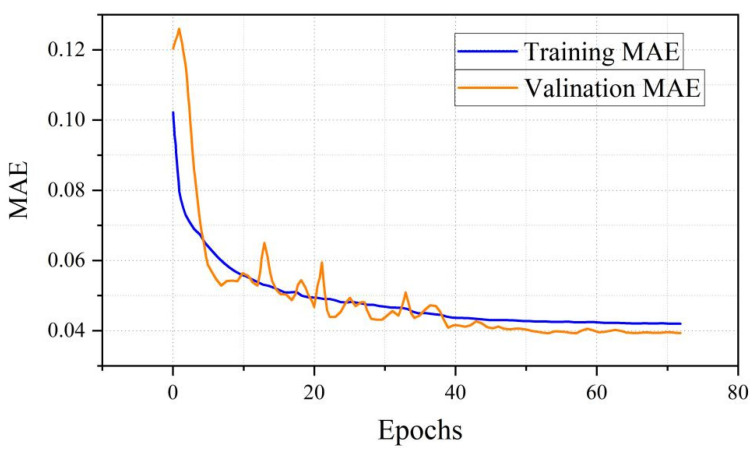
MAE Graph over 73 Epochs.

**Figure 9:**
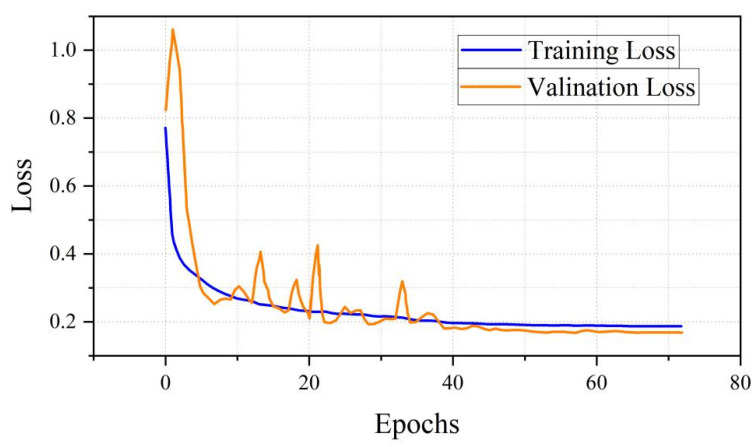
Loss Graph over 73 Epochs.

In this study, we employ a 1D-CNN architecture specifically designed for processing 1D data to identify mixed-material BRDF values. The optimal parameters used for this 1D-CNN identifier are summarized in Table 3. As shown in Figure 8, the model achieved a mean absolute error of less than 4.2% during the training phase and below 4.0% during the validation phase. Figure 9 depicts the evolution curves of the loss function throughout the training and validation processes.

The model exhibits exceptional performance in predicting the constituent proportions derived from various mixed-material BRDF signatures, which holds significant implications for practical fault diagnosis. To validate the predictive accuracy of the model, multiple sets of mixed-material BRDF data were constructed by combining the eight aforementioned materials in varying proportions. Calculations based on the experimental results indicate an overall maximum relative percentage error of 6.21%, corresponding to an overall prediction accuracy exceeding 93.79%. Detailed prediction data for specific materials are presented in Table 4.

## 4. Conclusions and Discussion

Addressing the critical scientific challenges of “data scarcity” and “inversion complexity” in space-based unresolved Space Object Identification, this paper conducts a systematic study ranging from high-precision experimental measurement to machine learning-based feature inversion. First, an automated BRDF measurement system was designed, operating within the 400–900 nm spectral range (with a spectral resolution better than 5 nm). Using an absolute measurement method, systematic BRDF characterizations were performed on eight common spacecraft materials. Comparative analysis of the resulting data facilitated the establishment of a laboratory database for aerospace material surface scattering. Second, this study validated the scientific hypothesis that the spectral-angular coupling features of mixed-material surfaces possess separable attributes. A machine learning model based on a One-Dimensional Convolutional Neural Network was proposed to process mixed-material BRDF data. The model was trained and validated to predict the constituent proportions within new mixed-material samples. Evaluation results demonstrate that the model achieved a prediction accuracy of 93.79% (corresponding to a maximum relative percentage error of 6.21%). In-depth research into the BRDF characteristics of aerospace materials holds urgent engineering significance for constructing dynamically updated space object feature libraries and optimizing space-based surveillance systems. Furthermore, it serves as a scientific prerequisite for overcoming bottlenecks in weak target detection and intelligent identification in complex environments, providing critical algorithmic models and physical data support for next-generation Space Situational Awareness and autonomous identification systems.

However, bridging the gap from “static laboratory measurements” to “on-orbit dynamic feature observation” in the process of Space Object Identification remains subject to the following scientific challenges: (1) Geometric and Dynamic Complexity: The BRDF data of space objects is influenced by physical shape, size, and attitude variations. On-orbit targets typically possess complex geometries and are often accompanied by dynamic characteristics such as rotation or tumbling. Consequently, the spectral curves observed on-orbit represent a non-linear superposition of material properties, geometric shapes, and mutual shadowing effects. Nevertheless, the laboratory data provided in this study remains the physical core constituting this complex integrated signal, as the key features of the total spectral curve are fundamentally determined by the BRDF properties of the surface materials. In addition, due to the energy demands of spacecraft, the solar panels of a normally operating satellite are generally oriented stably towards the sun. Therefore, we can utilize such pointing characteristics and the distinct spectral features of the solar panels to estimate the power consumption and volume of space targets. (2) Boundary Effects: Mutual shadowing and secondary scattering (inter-reflection) at the boundaries of surface materials may lead to deviations from the linear BRDF mixing assumption. However, for artificial satellites, the internal surface area of a material patch significantly exceeds the boundary area. Therefore, non-linear factors at the edges account for a negligible proportion of the total radiant flux. (3) Space Weathering: The complex space environment exerts unpredictable impacts on target surfaces. Long-term exposure to ultraviolet radiation and high-energy particle bombardment alters the microscopic surface roughness of materials, thereby changing their optical characteristics. The current database primarily characterizes pristine materials. Future work will involve simulating the space irradiation environment in the laboratory to measure the BRDF evolution of materials at different aging stages, thereby extending the “static” database into a “dynamic evolutionary database” incorporating the temporal dimension. (4) Illumination Variability: In actual space-based observations, illumination conditions are primarily determined by the solar phase angle but may also be affected by Earth albedo. However, such environmental influences do not alter the intrinsic waveform features of the material’s BRDF. The 1D-CNN model employed in this study, through normalization preprocessing and local convolution operations, effectively filters out global intensity fluctuations caused by environmental lighting. This allows for the efficient extraction of local morphological features determined by material properties, enabling robust inversion of mixed-material compositions.

## Figures and Tables

**Table 1:** Summary of the proposed model.

Layer	Output Shape	Number of Parameters
Conv1d	(51, 64)	384
Conv1d	(25, 128)	24,704
Conv1d	(12, 256)	98,560
Conv1d	(6, 512)	393,728
Conv1d	(6, 512)	786,944
Global Average Pooling	512	0
Dense	256	131,328
Dense	128	32,896

**Table 2:** Key statistical measures of the BRDF values for the eight materials.

Material	Minimum Value	Maximum Value	Scope	Trend
Aluminized Polyimide Film	2.99	23.55	20.56	It increases significantly with the wavelength, from approximately 3.0 (at 400 nm) to 23.6 (at 900 nm)
FEP	15.46	30.03	14.57	It increases significantly with the wavelength, from approximately 15.5 (at 400 nm) to 30.0 (at 900 nm)
Double-sided Aluminized PET Film	19.90	22.01	2.11	It decreases slightly with increasing wavelength, but the change is minimal (within the range of 21.0 to 22.0), indicating overall stability
Anodized Aluminum	0.1	2.34	2.24	It decreases from 2.3 (at 400 nm) to 0.1 (at 500 nm) and then increases slowly to 1.1 (at 900 nm), exhibiting an overall weak negative correlation
Black Painted Aluminum	1.69	3.62	1.93	It decreases gradually with increasing wavelength, from 3.6 at 400 nm to 1.7 at 900 nm
White Painted Aluminum	1.90	13.14	11.24	It decreases significantly with increasing wavelength, from 13.1 at 400 nm to 2.0 at 900 nm
Solar Panel	0.08	1.04	0.96	The values are relatively low, showing no distinct trend; they remain largely stable overall but with a slight upward tendency
Circuit Board	2.31	3.46	1.15	It increases gradually with increasing wavelength, from 2.3 at 400 nm to 3.5 at 900 nm

**Table 3:** 1D-CNN Parameters.

Parameter	Value
Optimizer	Adam
Activation Function	ReLU
Dropout	0.3 (Conv1d), 0.5 (Dense)
Epochs	73

**Table 4:** Prediction Accuracy for the Proportion of Each Component Material.

	Aluminized Polyimide Film	FEP	Double-Sided Aluminized PET Film	Anodized Aluminum	Black Painted Aluminum	White Painted Aluminum	Solar Panel	Circuit Board
Minimum Prediction Accuracy (%)	91.4	92.52	93.62	92.47	95.78	94.3	94.34	95.91
Maximum Relative Percentage Error (%)	8.53	7.48	6.38	7.63	4.22	5.7	5.66	4.09
Average Prediction Accuracy (%)	94	94.96	96.79	93.56	98.29	96.17	96.86	97.99
Average Relative Percentage Error	6	5.04	3.21	6.44	1.71	3.83	3.14	2.01

## Data Availability

Data are contained within the article.

## Abbreviations

The following abbreviations are used in this manuscript:

BRDF	bidirectional reflectance distribution function
1D-CNN	one-dimensional convolutional neural network
FEP	Fluorinated ethylene propylene
CCD	charge coupled device
